# Studying how state health services delivery policies can mitigate the effects of disasters on drug addiction treatment and overdose: Protocol for a mixed-methods study

**DOI:** 10.1371/journal.pone.0261115

**Published:** 2021-12-16

**Authors:** Matthew D. Eisenberg, Alexander McCourt, Elizabeth A. Stuart, Lainie Rutkow, Kayla N. Tormohlen, Michael I. Fingerhood, Luis Quintero, Sarah A. White, Emma Elizabeth McGinty

**Affiliations:** 1 Department of Health Policy and Management, Johns Hopkins Bloomberg School of Public Health, Baltimore, Maryland, United States of America; 2 Department of Mental Health, Johns Hopkins Bloomberg School of Public Health, Baltimore, Maryland, United States of America; 3 Division of Addiction Medicine, Johns Hopkins University School of Medicine, Baltimore, Maryland, United States of America; 4 Carey Business School, Johns Hopkins University, Baltimore, Maryland, United States of America; UNITED KINGDOM

## Abstract

**Background:**

The United States is experiencing a drug addiction and overdose crisis, made worse by the COVID-19 pandemic. Relative to other types of health services, addiction treatment and overdose prevention services are particularly vulnerable to disaster-related disruptions for multiple reasons including fragmentation from the general medical system and stigma, which may lead decisionmakers and providers to de-prioritize these services during disasters. In response to the COVID-19 pandemic, U.S. states implemented multiple policies designed to mitigate disruptions to addiction treatment and overdose prevention services, for example policies expanding access to addiction treatment delivered via telehealth and policies designed to support continuity of naloxone distribution programs. There is limited evidence on the effects of these policies on addiction treatment and overdose. This evidence is needed to inform state policy design in future disasters, as well as to inform decisions regarding whether to sustain these policies post-pandemic.

**Methods:**

The overall study uses a concurrent-embedded design. Aims 1–2 use difference-in-differences analyses of large-scale observational databases to examine how state policies designed to mitigate the effects of the COVID-19 pandemic on health services delivery influenced addiction treatment delivery and overdose during the pandemic. Aim 3 uses a qualitative embedded multiple case study approach, in which we characterize local implementation of the state policies of interest; most public health disaster policies are enacted at the state level but implemented at the local level by healthcare systems and local public health authorities.

**Discussion:**

Triangulation of results across methods will yield robust understanding of whether and how state disaster-response policies influenced drug addiction treatment and overdose during the COVID-19 pandemic. Results will inform policy enactment and implementation in future public health disasters. Results will also inform decisions about whether to sustain COVID-19 pandemic-related changes to policies governing delivery addiction and overdose prevention services long-term.

## Introduction

Disasters and the drug addiction and overdose epidemic are among the foremost public health issues facing the United States. Public health disasters, defined by the U.S. Centers for Disease Control and Prevention (CDC) as events that lead to a major disruption in health services [1], are predicted to increase in the coming decades due to climate change and other sources of geopolitical instability [2–4]. At the same time, the U.S. faces an ongoing drug addiction and overdose crisis. In 2019, one in five Americans aged 12 years or older used drugs non-medically; 8.3 million people met criteria for a substance use disorder involving drugs other than alcohol, and 70,630 died of a drug overdose [5, 6]. Provisional drug overdose death data for 2020 and 2021 show a sharp increase in overdose deaths spanning the COVID-19 pandemic [5], which has increased risk factors for drug use and overdose including psychological distress, poverty, unemployment, housing instability, and disruption of social networks [7–10].

Public health disasters pose major threats to addiction treatment delivery, and even brief treatment disruptions can prompt a return to drug use and attendant risk of overdose [11–15]. Relative to other types of healthcare services, addiction treatment is particularly vulnerable to disaster-related disruptions, for several reasons. People with drug addiction are disproportionately affected by socioeconomic barriers to treatment, such as poverty, unemployment, and housing instability [16–20], which may be exacerbated during disasters. In addition, drug addiction treatment is tightly regulated, which limits flexibility during disasters [7, 21–24]. For example, under federal law, methadone to treat opioid use disorder can only be dispensed in specialty clinics, which most patients must attend every day to receive their dose [25]. Addiction treatment is often disconnected from the general medical system, where critical on-the-ground disaster preparedness and response occurs; this fragmentation is a barrier to health system efforts to enhance addiction treatment access during disasters [26–29]. Finally, drug addiction treatment is stigmatized and under-resourced [30–36]. In disaster scenarios, which stretch health system resources and force choices about which services to prioritize, addiction treatment may be a low priority. All these factors also apply to overdose prevention services, such as distribution of naloxone or fentanyl testing strips by state and local public health departments.

Policies form the backbone of disaster response by delineating what health systems can and cannot do in the midst and aftermath of disasters. Most disaster policymaking occurs at the state level and is implemented at the local level by healthcare systems and local public health authorities [37–41]. The federal government’s role is primarily to allocate additional resources and provide authorization to make certain policy changes [40]. For example, in response to COVID-19, the federal government relaxed rules around in-person methadone dosing for opioid use disorder, allowing states to apply for a waiver to let clients take home a 14–28 days’ supply; some states opted to use that federal waiver, while others did not [42].

Several types of state health services delivery policies have the potential to enhance access to drug addiction treatment and prevent overdose during and after disasters, including state telehealth and Medicaid policies, state essential service designations, and policies explicitly targeting drug addiction treatment (Table 1). To date, however, these policies’ effects on drug addiction treatment and overdose during and following public health disasters have not been well studied. In addition, no prior studies have comprehensively considered strategies for effective implementation of state policies designed to mitigate disruptions to addiction treatment and overdose prevention services during public health disasters. The effectiveness of state policies depends upon local-level implementation because local healthcare systems and public health departments are the front-line of disaster response. Thus, it is critical to understand how local system-level policies and practices influence the implementation and effectiveness of the state-level policies of interest.

**Table 1:** State policies that may mitigate disruptions to health service delivery during/after disasters.

**PANEL A: State policies targeting health services generally**	**Hypothesized Mechanism**	**Outcomes**	**Population**
**State Telehealth Policies (“Telehealth” is defined as the provision of services remotely via internet or telephone technologies)**			
Telehealth coverage: state policies requiring that all services covered by insurance when delivered in-person must be covered via telehealth	These policies have been shown to increase physical health treatment via telehealth [63–68]. We expect such policies to enhance access to addiction treatment, which reduces risk of overdose [11, 69].	All types of addiction treatment delivered via telehealth, fatal & non-fatal overdose	Medicaid, fully insured commercial insurance beneficiaries
Telehealth reimbursement: state policies requiring that insurance reimbursement rates must be identical for in-person/telehealth services			
No in-person relationship rule: state policies stating that providers can treat new patients via telehealth without in-person consultation			
**State Medicaid Policies**			
Prior authorization suspension: state policies suspending prior authorization (i.e., pre-approval of services by insurer) requirements for all services	These policies remove barriers to treatment shown to impede treatment access [7–73]. Addiction treatment reduces risk of overdose.	All types of addiction treatment delivered in-person or via telehealth, fatal & non-fatal overdose	Medicaid beneficiaries
Cost sharing suspension: suspension of cost-sharing (e.g., co-payments)			
Renewal suspension: state policies suspending the Medicaid enrollment renewal requirement			
**PANEL B: State policies targeting addiction treatment, overdose**	**Hypothesized Mechanism**	**Outcome(s)**	**Population**
**State Addiction Treatment Telehealth Policies**			
Audio-only: state policies allowing addiction treatment to be delivered by phone	People with addiction are disproportionately low-income [16, 74, 75], may lack access to technology needed for telehealth via computer	All types of addiction treatment delivered via telehealth, fatal & non-fatal overdose	Medicaid, fully insured commercial insurance beneficiaries
Buprenorphine renewal: state policies allowing buprenorphine prescriptions to be renewed via telehealth	These policies allow access to buprenorphine without requiring an in-person visit; buprenorphine treatment reduces overdose	Buprenorphine treatment for OUD delivered via telehealth, fatal & non-fatal overdose	Medicaid, fully insured commercial insurance beneficiaries with OUD
Buprenorphine initiation: state policies stating that buprenorphine for opioid use disorder can be initiated for the first time via telehealth			
**State Essential Service Designations**			
Addiction treatment services designated essential: state policy designating specialty addiction treatment programs as essential services	Essential services stay open during the disaster	All types of addiction Tx	Everyone; insured, uninsured
Harm reduction services designated essential: state policy designating harm reduction programs, such as syringe services programs, as essential		Fatal overdose	
**Other Policies**			
Take-home methadone waiver: state fully adopted waiver allowing 14–28 days’ take-home supply for all people using methadone to treat opioid use disorder	This policy relaxes in-person dosing rules; methadone treatment reduces overdose [11]	Methadone for OUD, fatal & non-fatal overdose	Everyone; insured, uninsured
Policies Enhancing Harm Reduction Service Delivery: policies relaxing limits on the amount of harm reduction supplies (naloxone, fentanyl testing strips) organizations can distribute and how they can be requested (e.g., online) and distributed (e.g., home drop-off)	These policies support effective fatal overdose prevention services [76, 77]	Fatal overdose	Everyone; insured, uninsured

### Materials and methods

#### Aim 1

Study Aim 1 is to examine how state policies designed to mitigate the effects of the COVID-19 pandemic on health services delivery influenced receipt of addiction treatment. We will use a difference-in-differences analysis of insurance claims and addiction treatment program admissions data from the 50 U.S. states. We expect the policies of interest—all of which are designed to enhance health service access—to mitigate disruptions to addiction treatment during and following the COVID-19 public health disaster.

#### Aim 2

Study Aim 2 is to examine how state health services delivery policies influenced the effects of the COVID-19 crisis on fatal and non-fatal overdose. We will use the same difference-in-differences approach in Aim 1 to analyze insurance claims and mortality data. We expect that the policies of interest will mitigate increases in overdose during and following the COVID-19 disaster.

#### Aim 3

Study Aim 3 is to characterize local-level implementation of state health services delivery policies put in place in response to COVID-19 and to identify policy gaps and changes needed to enhance addiction treatment access and prevent overdose during and following public health disasters. In this Aim, we will conduct interviews with state and local policy implementation leaders and review relevant local government, healthcare system, and public health system policy documents. We will characterize local healthcare and public health system policies and practices designed to support access to addiction treatment and overdose prevention services during the COVID-19 disaster; consider how these local health system policies and practices may interact with state policies to influence on-the-ground implementation of policies enacted at the state level; and examine policy implementation leaders’ perceptions of policy and practice gaps and strategies to address them. We will also explore front-line policy implementers’ perceptions of how state health services delivery policies designed to mitigate disruptions to health services during the COVID-19 pandemic could generalize to other disasters and examine leaders’ perceptions of whether and how the state policies of interest, if sustained long-term, would enhance access to addiction treatment and prevent overdose post-COVID-19 pandemic.

## Methods

### Study design and sample

The study uses a concurrent-embedded design. Aims 1 and 2 quantitative analyses guide the research, and the secondary Aim 3 qualitative study plays a supportive role. Quantitative Aims 1–2 use a difference-in-differences design to analyze the effects of policies on outcomes using secondary data sources that include data on all 50 U.S. states. Qualitative study Aim 3 uses an embedded multiple case study approach, in which we characterize policy implementation in counties embedded within states. Aims 1–3 will be conducted concurrently.

### Study period

The study period is 2015–2023, encompassing the five years prior to the COVID-19 pandemic and four years following onset on the pandemic in the U.S. Aims 1–2 quantitative analyses will include data for each of the nine years in the study period. The Aim 3 qualitative study will primarily focus on implementation of state health services delivery policies put in place in response to the COVID-19 pandemic. Aim 3 will also characterize local disaster preparedness policies that were in place pre-COVID and explore leaders’ perceptions of how those pre-pandemic policies influenced COVID-19 disaster response.

### Data sources

#### State health services delivery policy data

Data on the state health services delivery policies of interest in Table 1 will be assembled by the public health lawyers on the study team using legal research and legislative history techniques [43], including full-text searches of the Westlaw database and identification of state executive orders, session laws, regulatory materials, and sub-regulatory guidance. The data will include each policy’s provisions, effective date, and end date (if applicable). While many policies put in place in response to the COVID-19 disaster were designed to be temporary, as of November 2021, there are ongoing policy dialogues to consider making some of these policy changes permanent, as they have the potential to enhance service access long-term [44–47].

#### Administrative claims data

Aims 1–2 will use IQVIA and OptumLabs^®^ Data Warehouse (OLDW) administrative claims to measure addiction treatment (Aim 1) and overdose-related treatment utilization (Aim 2) outcomes. The IQVIA data captures 93% of all U.S. retail prescriptions, as well as outpatient services delivered by ≈75% of U.S. licensed physicians. Using a portal embedded in their billing software, pharmacies and outpatient clinics generate daily data that is automatically transmitted to IQVIA. A key strength of the IQVIA data is that it captures services from all payers, including services paid by any insurer (e.g., commercial insurance, Medicaid, Medicare) or by cash. The OLDW contains de-identified administrative claims data, including medical and pharmacy claims on commercial insurance enrollees and patients, representing a diverse mixture of ages, ethnicities and geographical regions across the United States [48]. Both data sources include claims for patients of all ages and have information on patient age, sex, state, 5-digit zip-code and medications, diagnoses, and procedures received, along with dates of receipt. The IQVIA and OLDW data are complementary in that the OLDW data includes inpatient and emergency department claims as well as a flag to identify commercial insurance beneficiaries in fully insured plans (as noted in Table 1, some policies of interest apply to this subset of beneficiaries), information not included in the IQVIA data.

#### Specialty addiction treatment program admissions data

Aim 1 will use Treatment Episode Data Set (TEDS) admissions data to measure inpatient and ambulatory admissions to specialty addiction treatment facilities. The data capture two-thirds of specialty addiction treatment programs in the U.S., including programs in all 50 U.S. states, and contain records on those aged 12 or older. TEDS includes clinical characteristics (e.g., substances used, frequency of use) as well as information regarding the date and type of treatment admission (e.g., detoxification, intensive outpatient). Patient demographic information includes age, sex, race/ethnicity, state of residence, and core-based statistical area (CBSA) of admission. The TEDS data is complementary to the claims data sources described above: as addiction treatment is often delivered outside the general medical sector and paid for through federal and state grant programs, the specialty programs included in TEDS are underrepresented in the claims data.

#### Mortality data

Aim 2 will use CDC multiple cause of death data to measure fatal overdoses. The data includes underlying cause of death, state, zip-code, and decedent demographics including age, sex, and race/ethnicity. Where the administrative claims data captures treated overdose episodes—many of which are non-fatal—the mortality data captures all fatal overdoses, including those that occur outside of the healthcare system.

#### Mobility data

Measuring mobility is critical to disentangling the effects of the state health services delivery policies of interest from the effects of COVID-19-related disruptions to in-person health service delivery on outcomes. We will use cell phone tracking data to measure area-level mobility. This data comes from signals, or ‘pings,’ that identify the location of smartphones at a moment in time, and includes information about duration, origin, and destination of trips made by people with smartphones, including specific information on points of interest (e.g., healthcare settings) visited.

#### Area-level characteristics data

Aims 1–2 will also use data on area-level characteristics from the U.S. Census Bureau data including rural/urban status, racial/ethnic, income, education, and home ownership distribution, data from the Area Health Resource File on health provider density, and data from the National Survey of Substance Abuse Treatment Services (N-SSATS) on specialty addiction treatment program density.

#### Qualitative data

Aim 3 qualitative data will be collected through semi-structured interviews and document collection. In the states and counties included in the Aim 3 sample (see section below), we will recruit state-level, county-level, and healthcare and public health system-level leaders in addiction treatment, overdose prevention, and disaster preparedness and response. The interview guide will open with an overview of the study and an opportunity to ask questions, followed by innocuous and grand tour questions to establish rapport, and then by researcher-driven questions [49–55]. The researcher-driven portion of the interview guide will be structured in two sections: a state policy section and a local policy and practices section. In the state policy section, interview guide domains will include perceived importance and feasibility of the specific state health services delivery policies put in place in an interviewees’ state in response to the COVID-19 pandemic; policy implementation strategies, barriers, and facilitators; perceptions of policy gaps and needed changes; views on how policies used in COVID-19 generalize to other disasters; and thoughts on whether and how policies should be sustained long-term. The local policy section of the guide will be focused on identifying local healthcare and public health system policies and practices put in place to support health services delivery during COVID-19 and exploring leaders’ perceptions of how these local policies interact to support or impede implementation of the state-level policies of interest in Aims 1–2.

The semi-structured interview guide will be developed by the study team in close consultation with the study’s advisory board, which includes experts in addiction treatment, overdose prevention, healthcare administration, and health system disaster preparedness and response, as well as individuals with lived experience of drug addiction during the COVID-19 pandemic. Interviews will be conducted by a single master’s-level study team member trained in qualitative interviewing, via videoconference. Table 2 provides additional details regarding our qualitative research methods within the Consolidated Criteria for Reporting Qualitative Studies (COREQ) framework. We will ask interviewees to provide supplementary documentation regarding COVID-19 disaster response in their jurisdiction, e.g., state or local public health department disaster response memoranda or healthcare system emergency management plans. We will identify additional publicly available policy documents by searching local system websites.

**Table 2:** Qualitative study design.

**Research Team and Reflexivity**	
Personal Characteristics	
1. Interviewer/facilitator	All interviews will be conducted by the same member of the study team.
2. Credentials	The interviewer will be a masters-level trained researcher.
3. Occupation	The interviewer will be employed full-time as a research associate.
4. Gender	The interviewer will be female.
5. Experience and training	The interviewer will have experience participating in qualitative research studies and will be supervised by the study PI, who has extensive training and experience conducting qualitative research.
Relationship with Participants	
6. Relationship established	Potential interviewees will be contacted with a standardized recruitment email to introduce the study and the interviewer and to request their participation.
7. Participant knowledge of the interviewer	The recruitment email will explain the study goals and why the interviewer is interested in conducting this research. This information will be reviewed at the start of each interview.
8. Interviewer characteristics	The recruitment email will provide information about the research team, including the interviewer. This information will be reviewed at the start of each interview.
**Study Design**	
Theoretical Framework	
9. Methodological orientation and theory	The qualitative portion of the study will use a content analysis approach.
Participant Selection	
10. Sampling	Potential interviewees will be selected based on their professional roles related to the policies of interest.
11. Method of approach	Potential interviewees will be approached with a standardized recruitment email.
12. Sample size	We anticipate conducting 12–15 interviews in each of the 8 intervention states.
13. Non-participation	We will document any reasons provided by those who decline to participate as well as any individuals who do not respond to our recruitment email.
Setting	
14. Setting of data collection	Data will be collected via interviews conducted by videoconference or, if not feasible, by telephone.
15. Presence of non-participants	We anticipate that the interviewer and interviewee will be the only individuals present.
16. Description of sample	The sample will include key implementation leaders for the policies of interest in the two states with the highest per-capita COVID-19 death rate in each of the four U.S. census regions (eight states total).
Data Collection	
17. Interview guide	The interview guide will be developed by the study team and shared with an advisory board for feedback. It will be pilot tested and refined before data collection begins.
18. Repeat interviews	We will conduct repeat member-checking interviews with a random sample of 20–30 interviewees.
19. Audio/visual recording	Once permission is granted, videoconference/telephone interviews will be recorded.
20. Field notes	The interviewer will draft summary notes immediately after concluding each interview.
21. Duration	We anticipate that interviews will last no more than 90 minutes.
22. Data saturation	The study team will convene on a regular basis to review interview data and determine when data saturation is reached. Saturation will be defined as no new key themes arising from the data.
23. Transcripts returned	We do not plan on returning transcripts to interviewees. Based on the straightforward nature of our questions and prior research with similar types of interviewees, we do not anticipate that this will be necessary.
**Analysis and Findings**	
Data Analysis	
24. Number of data coders	We plan to have two coders pilot a sub-sample of transcripts. Once discrepancies are resolved and the codebook is finalized, the full set of transcripts will be coded by one individual.
25. Description of the coding tree	We plan to develop a coding tree (i.e., codebook) based on a review of the literature, a priori knowledge within the study team, and summary notes from interviews. We will also share a draft codebook with our advisory board for feedback.
26. Derivation of themes	Themes will be derived once data have been coded. Preliminary themes may be identified based on discussions with the interviewer and review of field notes.
27. Software	We plan to use NVivo qualitative research software.
28. Participant checking	A bulleted list of key findings will be shared with participants once data have been coded and analyzed.
Reporting	
29. Quotations presented	Quotations from interviews will be used to present findings, and they will be accompanied by an interviewee identification number.
30. Data and findings consistent	Our planned use of quotations will allow for assessment of consistency between our data and findings. We will also create supplemental tables with additional quotations to share as much information as possible when presenting our findings.
31. Clarity of major themes	We plan to use sub-headings listing our major themes to promote clarity when writing up our findings.
32. Clarify of minor themes	We plan to provide quotations from interviewees who raised minor themes or shared information contrary to findings of our major themes.

### Study sample

Aims 1–2 analytic samples will include data from all 50 U.S. states and Washington, D.C. Aims 1–2 claims data analyses will use continuous cohorts of patients of all ages diagnosed with a substance use disorder during the 2015–2023 study period, with estimated sample sizes of 24,000,000 individuals in the IQVIA data and 475,000 individuals in the OLDW data. The Aim 1 analysis of specialty drug treatment admissions will be conducted using all admissions among people aged 12 years or older included in the TEDS data from 2015–2023, with an estimated sample size of 8,000,000 admissions. The Aim 2 overdose analyses will be conducted using all overdose deaths in the OLDW claims (same estimated sample size as above) and CDC mortality data (estimated sample size 644,000 drug overdose deaths) from 2015–2023. As shown in Table 1, analyses of policies applying to specific subsets of individuals (e.g., Medicaid beneficiaries) will be limited to those groups. The Aim 3 study sample will include eight states: the two states with highest per-capita COVID-19 death rate in each of the four U.S. census regions at the start of the qualitative study. Within each of those states, we will conduct embedded case studies of two counties: the urban county with the largest population and the rural county with the largest population (16 counties total). We expect to interview approximately 115 total state and local-level policy implementation leaders. Interviews will be conducted until data saturation, defined as no new key themes emerging from the data, is reached.

### Measures

#### Aims 1–2

Final analytic measures will be constructed at the person-month (or admission-month in the TEDS data, which identifies admissions rather than individuals) level in Aim 1 and state-month level in Aim 2. Aims 1–2 independent variables are binary indicators of the state health services delivery policies of interest, which change from zero to one starting the first state-month a policy is enacted.

Aim 1 dependent variables include measures of receipt of any inpatient, ED, or outpatient drug addiction treatment service (claims data sources); any receipt of a prescription for a medication used to treat opioid use disorder (claims data sources); and any specialty drug addiction treatment admission (TEDS). In claims data analyses, among people who receive any of these services, we will also measure the number of services received. Aim 2 dependent variables include measures of fatal and non-fatal drug overdose rate per 100,000 population.

To measure area-level mobility in Aims 1–2, we will use the cell phone data to create measures of the ratio of the volume of travel in a person’s zip-code (CBSA in TEDS) in each month relative to the same month in 2019, pre-COVID-19 pandemic. We will construct separate measures of overall mobility and traffic to healthcare settings. In Aims 1–2 we will also construct zip-code/CBSA-level measures of urban/rural, racial/ethnic, income, education, and home ownership distribution, as well as state-level measures of physician and specialty addiction treatment program density. For Aim 2, where final analytic measures will be constructed at the state-month level, we will link zip-code/CBSA data in the granular overdose-level data and then ‘roll-up’ the data to the state-month level, e.g., the percent of people in a given state-month who lived in a zip-code with 25%, 50%, or 75% mobility relative to the same most recent pre-COVID state-month.

#### Aim 3

Aim 3 qualitative interviews will yield key themes aligning with the interview guide domains described above. In addition, we will characterize local (e.g., county government, healthcare system, public health system) policies put in place to mitigate disruptions to health services generally or addiction services specifically in the eight states in the Aim 3 sample.

### Analysis

#### Aims 1–2

In Aims 1–2, we will use a difference-in-differences approach to compare trends in outcomes before and after the implementation of state health services delivery policies in states with versus without these policies. To illustrate the general model specification, the Aim 1 model for administrative claims data analyses—in which the unit of observation is person-months nested in zip-codes, which are nested in states—will take the following form:

$$y_{izst}=f(\alpha_{0}+\beta_{t}Policy_{st}+\phi Mobility_{zst}+X_{it-1}^{\prime }\delta +Z_{zt}^{\prime }\lambda +P_{st}^{\prime }\pi +\gamma_{t}Month_{t}+\theta_{s}State+\varepsilon_{izst})$$

where *y*_*ist*_ is one of the outcomes of interest, *Policy*_*st*_ is an indicator variable equal to one if state *s* had the health service delivery policy of interest in effect during time *t*, *Mobility*_*zt*_ is the overall level of mobility in individual’s *i*’s zip code during time *t*, *X*_*i*,*t-1*_ is a vector of individual-level pre-period covariates, *Z*_*zt*_ is a vector of zip code-level covariates, *P*_*st*_ is a vector of state-level provider availability measures, *Month*_*t*_ are a series of fixed effects for each calendar month, and *State*_*s*_ is a vector of state fixed effects.

To address the multilevel nature of the data, we will calculate standard errors via the delta method and employ two-way clustering at the zip-code-state level [56, 57]. Aim 1 TEDS data analyses and Aim 2 analyses follow a conceptually similar form. In Aim 1 analyses of the TEDS specialty addiction treatment program admission data, the unit of analysis is admission-month and rather than zip-code we use CBSA, the most granular geographic identifier in TEDS. In Aim 2, where the unit of analysis is state-month, the mobility measure and all area-level covariates will be aggregated to the state level, and we will cluster standard errors by state. In both Aims 1–2, area-level characteristic measures will be employed as either covariates, as depicted in the model specification above, or effect modifiers. For example, we will conduct effect modification analyses to examine whether the effects of the state health services delivery policies differ in urban versus rural areas.

We will also consider multiple conceptualizations of the overall and healthcare setting-specific mobility measures. Over the course of the COVID-19 pandemic, mobility has varied across states and localities, and within jurisdictions over time, due to spiking/waning of COVID-19 cases; in response to jurisdictions imposing, lifting, and reinstating physical distancing rules; and as virus fatigue has set in and led some people to increase their mobility irrespective of case rates or policy mandates. In a difference-in-differences framework, covariates are defined as variables that vary by treatment group and could cause variation in outcome trends over time. Given variation in mobility across states and the fact that mobility could influence our treatment and overdose outcomes of interest, mobility could operate as a covariate. We will also examine mobility as an effect modifier, e.g., by assessing whether policy effects differ in areas with low versus high mobility. In addition, we will explore the possibility that mobility to healthcare settings could be on the causal pathway between the state policies of interest and outcomes by examining the relationship between the policies and mobility during the pandemic as a dependent variable. For example, in a scenario where a healthcare system offers both in-person and telehealth visits, a state policy offering enhanced coverage of telehealth services could lead some people to choose telehealth, leading to a lower level of traffic to healthcare settings than would be observed if the policy were not in place.

Given that some states implemented multiple policies of interest at the same time, we will analyze the effects of individual policies as well as combined effects of grouped policies, e.g., at least one telehealth policy in Table 1, all telehealth policies, etc. Our ability to make causal inferences about specific policies/groups of policies is enhanced by the fact that different policies are expected to effect different populations and outcomes (Table 1). Recent work shows that in scenarios where policy adoption is staggered across geographic units, traditional difference-in-differences specifications with two-way state and time fixed-effects, like the model above, can produce biased results. While adoption of the state health services delivery policies of interest consistently occurred in March or early April 2020, there may be staggered policy phase-out across states. If this is the case, as identified through our legal mapping, we will analyze treatment heterogeneity following Goodman-Bacon 2021 [58] and, as necessary, employ alternative approaches to address two-way fixed-effects-related biases [59–62].

#### Aim 3

After each interview, we will create summary memos identifying preliminary themes. These memos, along with the interview guide, will contribute to the development of a codebook. Using a randomly selected sub-sample of transcripts, two team members will pilot the codebook. It will then be further refined and analyzed with input from the advisory board. The final codebook will be applied to all interview transcripts. Text segments will be organized in QSR NVivo v11 and analyzed according to themes and sub-themes. We will compare themes across states and counties, for example to identify varying patterns in themes in urban versus rural counties. We will identify local healthcare and public health system policies of interest through combined analyses of interviews (when we will ask interviewees to identify policies) and policy documents. We will conduct member-checking with a random sample of interviewees who will review key themes.

### Ethical considerations

Aims 1–2 of this study involve analysis of secondary data sets that meet limited data set criteria and do not include any individual identifying information such as name or medical record number. Aim 3 involves qualitative interviews with state and local health system leaders and focuses on topics related to their professional roles. The study was deemed exempt by the Johns Hopkins Bloomberg School of Public Health Institutional Review Board on May 11, 2021 (IRB #16400). Consent was waived for the Aims 1–2 limited data sources. Oral consent will be obtained from participants in qualitative interviews.

### Status and timeline of the study

As of November 2021, participant recruitment and data analyses have not yet begun. These activities will begin in winter 2022. Aims 1–2 secondary data analysis will be conducted iteratively from 2022–2025 as the latter years of data included in the study become available. Aim 3 qualitative data collection will be conducted over an approximately 15-month period beginning in winter 2022. The study will be completed by 2026.

## Discussion

This study will provide important insights into the implementation and effects of state health services delivery policies on addiction treatment and overdose during the COVID-19 pandemic. Aim 3 qualitative interview and document review results will inform the final design and interpretation of Aims 1–2 differences-in-differences analyses. For example, if we find in Aim 3 that interviewees’ view a given type of state policy as having effects on outcomes in urban but not rural areas, we could conduct an analysis limiting the sample in the difference-in-differences analysis to residents of urban counties. If we find consistently minimal local-level implementation of a given type of state policy in the 16 counties included in the Aim 3 case study, that may help to explain null findings for that policy in Aims 1–2. In addition, the Aim 3 qualitative study is designed to identify disaster-response health services delivery policy implementation best-practices, gaps, and strategies to overcome those gaps, as well as insight into whether and how the state policies evaluated in Aims 1–3 could generalize to other types of public health disasters. This study provides an example of a mixed-methods design supplementing econometric policy evaluation results with qualitative data characterizing details about policy implementation that are highly relevant to decision-makers and front-line policy implementers.

A key contribution of this study is consideration of local implementation of state public health policies. As in Aims 1–2 in the study described in this protocol, quantitative policy evaluations typically assess a policy of interest at a single level, e.g., state laws. However, on-the-ground policy implementation is often influenced by policies and practices at more granular levels. The disaster-response health services delivery policies in this study are an excellent example of this phenomenon, in that state policies must be implemented within counties and cities by local public health and healthcare systems. Policies and practices within those local jurisdictions and systems—for example, county-level designations of what services are deemed “essential” and can therefore be delivered in-person during disasters, a healthcare system policy delineating staffing for a virtual addiction consult service during the COVID-19 pandemic, or a local public health department policy laying out procedures for filling online requests for naloxone during the pandemic—likely influence implementation of state-level policies. Our study is designed to characterize policy implementation leaders’ perceptions of how these local factors influence implementation of the state health services delivery policies of interest. This analysis could be hypothesis-generating and inform the design of future quantitative studies assessing how state-local disaster-response policy interactions influence addiction treatment and overdose—or other health services and outcomes—during public health disasters.

A limitation of the study described in this protocol is our inability to measure policies’ effects on overdose prevention service delivery. No comprehensive data on delivery of these services exists. In a sensitivity analysis, we will explore policies’ effects on receipt of naloxone prescriptions in the IQVIA and OLDW data; however, most naloxone is distributed by health departments and other entities under standing orders with no prescriptions. To address the multi-level nature of our Aims 1 insurance claims data, we employ a two-way clustering of standard errors at the zip-code-state level. While our main approach uses fixed intercepts, which can be preferable for teasing out policy effects, we will also conduct sensitivity analyses using multi-level (hierarchical) modeling with random state and zip-code level intercepts. Another key consideration in this study is the complex policy environment in which states and localities implemented multiple policies designed to mitigate the effects of the COVID-19 pandemic on health services delivery at or around the same time. The study is designed to both acknowledge and unpack this complex environment by aligning specific policies with specific target populations and outcomes (e.g., state Medicaid policies should only affect Medicaid beneficiaries; state telehealth policies should only affect addiction treatment services delivered via telehealth technology) and by triangulating quantitative policy evaluation results from Aims 1–2 with in-depth qualitative data explaining front-line implementers’ perceptions of how policies work on the ground.

Little is known about how various state policies designed to mitigate the adverse effects of the COVID-19 pandemic on health services delivery have influenced drug addiction treatment and overdose. People who experience addiction and/or overdose risk are at particularly high risk of morbidity and mortality due to disaster-related service disruptions. Our study’s results will fill this gap and inform the design and implementation of policies in future public health disasters.
